# Computations of uncertainty mediate acute stress responses in humans

**DOI:** 10.1038/ncomms10996

**Published:** 2016-03-29

**Authors:** Archy O. de Berker, Robb B. Rutledge, Christoph Mathys, Louise Marshall, Gemma F. Cross, Raymond J. Dolan, Sven Bestmann

**Affiliations:** 1 Sobell Department of Motor Neuroscience and Movement Disorders, UCL Institute of Neurology, University College London, London WC1N 3BG, UK; 2 Wellcome Trust Centre for Neuroimaging, Institute of Neurology, University College London, London WC1N 3BG, UK; 3 Max Planck University College London Centre for Computational Psychiatry and Ageing Research, London WC1B 5EH, UK; 4 Clinical Biochemistry, King's College Hospital, Denmark Hill SE5 9RS, UK

## Abstract

The effects of stress are frequently studied, yet its proximal causes remain unclear. Here we demonstrate that subjective estimates of uncertainty predict the dynamics of subjective and physiological stress responses. Subjects learned a probabilistic mapping between visual stimuli and electric shocks. Salivary cortisol confirmed that our stressor elicited changes in endocrine activity. Using a hierarchical Bayesian learning model, we quantified the relationship between the different forms of subjective task uncertainty and acute stress responses. Subjective stress, pupil diameter and skin conductance all tracked the evolution of irreducible uncertainty. We observed a coupling between emotional and somatic state, with subjective and physiological tuning to uncertainty tightly correlated. Furthermore, the uncertainty tuning of subjective and physiological stress predicted individual task performance, consistent with an adaptive role for stress in learning under uncertain threat. Our finding that stress responses are tuned to environmental uncertainty provides new insight into their generation and likely adaptive function.

## Abstract (web summary)

Acute stress has broad physiological and behavioural consequences, yet the precise factors that generate stress responses are not known. Here, de Berker and colleagues demonstrate that acute stress responses dynamically track environmental uncertainty and predict ability to learn under uncertain threat.

## Graphical abstract

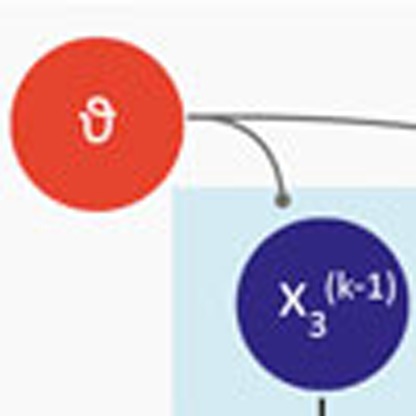

Stress has broad ranging physiological consequences^1^. Although acute stress is often characterized as a challenge to homeostasis, the precise features of the environment that contribute to the generation of stress responses are largely unknown. Understanding the computations that underlie acute stress responses is important for insight into how stress relates to adaptive behaviour, potentially illuminating links between stress and disease, and facilitating treatment of stress-related disorders^2^.

Extant work suggests that unpredictability and uncontrollability are central features of stressful experiences^3^,^4^,^5^,^6^,^7^. Classic experiments in rodents demonstrate that rats exposed to a series of electric shocks show attenuated stress responses if they are able to predict or control the timing of a stressor^8^, with subsequent work in humans documenting increased pain and stress in response to unpredictable stimuli^9^,^10^,^11^. However, such experiments typically contrast conditions of complete unpredictability to those of complete predictability^4^. Such binary comparisons do not capture the dynamic nature of uncertainty, which varies as an organism learns about and interacts with its environment^12^. Furthermore, stress affects learning^13^, suggesting that reduction of uncertainty may be a function of stress responses. Previous approaches have thus left several key questions unanswered.

First, it remains to be demonstrated whether the subjective and autonomic responses to acute stressors track fluctuations in uncertainty over time, which would imply a link between the processes underpinning learning and those of stress control. Second, it is unclear whether such responses relate to objective unpredictability or whether they are entrained to subjective estimates of uncertainty. If so, interindividual variation in assessment of uncertainty might provide insight into the considerable variation in acute stress responses across individuals^14^. Finally, recent work has demonstrated that individuals track separable forms of uncertainty during learning^15^, and it is unclear which form of uncertainty is important for driving subjective and autonomic responses to acute stressors^16^.

To address these questions, we here adopt a subject-specific Bayesian model of decision making to track distinct forms of uncertainty and examine their relationship to acute stress responses during an aversive learning paradigm (Fig. 1). Uncertainty can be parsed into several distinct forms^17^,^18^, for which there exists a variety of theoretical and neurobiological evidence^12^,^15^,^19^,^20^. For example, forecasters predicting the performance of a football team over the coming season face several distinct sources of uncertainty when formulating their predictions. First, there is ‘irreducible uncertainty', which captures a chance slip by a goalkeeper or a fortuitously mis-struck shot. Irreducible uncertainty reflects the randomness inherent to any complex environment. Irreducible uncertainty might increase if it begins to rain, decreasing the accuracy with which players move. Second, after a series of summer signings, it may be unclear how good a team is, producing uncertainty about the probability of a team winning each match. As the season progresses, this ‘estimation uncertainty' falls as the strengths and weaknesses of the team become evident, although it may vary with local dips and surges in form. A third source of unpredictability in this context is managerial instability. Assuming the manager influences performance, uncertainty about how long the current manager will remain in charge makes it harder to predict performance. This ‘volatility uncertainty' is about the stability of the context. To appreciate this, compare the stability in English football of Arsenal Football Club (1 manager for the last 18 years) to the famous volatility of Newcastle United (19 managers over the same period).

To dissect the role of these three forms of uncertainty in acute stress, we utilized an hierarchical Bayesian perspective^21^ (Fig. 2a). Importantly, the model is fit individually to each subject, with two free parameters (*ϑ* and *ω*) capturing variation between individuals and allowing for divergence between subjective and objective uncertainty. In this framework, beliefs at several levels of a probabilistic hierarchy are represented as Gaussian distributions characterized by means and variances, the latter quantities corresponding to uncertainty. This naturally captures the sources of uncertainty described above: irreducible uncertainty resulting from probabilistic relationships between predictors and outcomes, estimation uncertainty resulting from imperfect knowledge of those probabilistic relationships, and volatility uncertainty reflecting potential environmental instability^17^. How these different forms of uncertainty contribute to subjective and autonomic responses to acute stressors is unknown.

We evaluated the contribution of different forms of uncertainty to subjective and physiological stress responses, using a commonly employed acute stressor over which we had precise control, electric shock^22^,^23^,^24^,^25^. Participants completed a probabilistic learning task (Fig. 1) in which electric shocks were delivered with varying predictability. On each trial, a stimulus (rock A or rock B) was presented and participants were asked to predict whether or not there was a snake underneath (snake or no snake). Each time a snake was presented, participants received a painful electric shock to the hand. We used a computational model of learning^21^ (Fig. 2a) to estimate the dynamic fluctuations in uncertainty experienced by each individual based on the predictions they made. Although the sluggish dynamics of endocrine responses preclude a detailed analysis of the relationship between cortisol release and uncertainty^26^, we measured salivary cortisol levels by way of confirmation of our stress induction. We complemented this slow measure with high-frequency assessments of subjective stress and sympathetic arousal, which vary on a timescale equivalent to the forms of uncertainty in which we are interested. This allowed us to examine the online evolution of stress responses rather than merely their delayed endocrine consequences, which may have distinct determinants and function^27^,^28^. As acute stress involves the co-ordinated action of emotional, physiological and motivational systems^29^, we measured subjective stress ratings, pupil diameter and skin conductance (Fig. 1d) throughout the task. Pupil diameter and skin conductance provided established measures of activity in the autonomic nervous system, a key effector of acute stress responses^30^,^31^,^32^. We found that all three were predicted by subjective irreducible uncertainty. We further examined interindividual variance in the degree of coupling between uncertainty and stress responses, which we related to the ability of participants to learn in an uncertain dynamic environment.

## Results

### Unpredictable aversive threat induces stress

**Figure 1:**
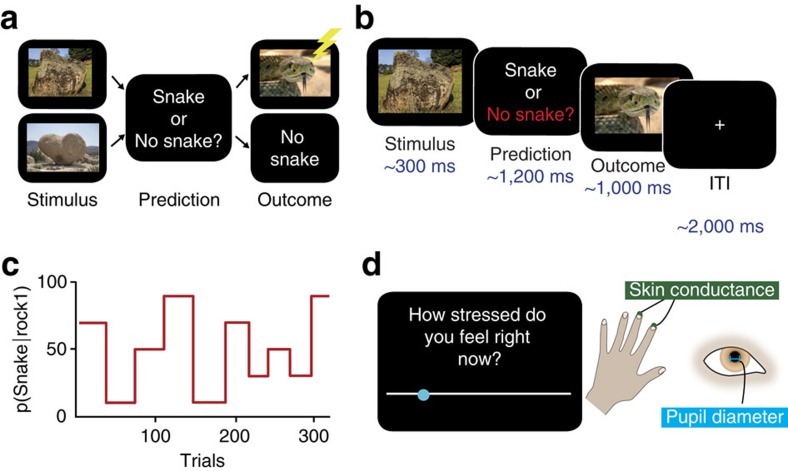
Task structure and stress measures. (**a**) Learning task. Visual stimuli (rocks) were probabilistically associated with outcomes (snake or no snake). Subjects made a prediction of the outcome on each trial. The appearance of a snake was accompanied by the delivery of a painful electric shock. (**b**) Example trial. Here the participant incorrectly predicts no snake. Timing was jittered; see Methods. (**c**) The probabilities governing stimulus–outcome relationships shifted unpredictably over time, producing fluctuations in uncertainty. (**d**) Subjective stress ratings were collected every four to six trials. Measures of skin conductance (*n=*45) and pupil dilatation (*n=*22) were collected in some subjects.

On each trial, participants (*n=*45) were shown one of two rocks and asked to predict whether or not there was a snake underneath (Fig. 1a,b). Participants were explicitly informed of the reciprocal probabilities linking the two stimuli:


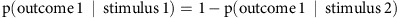


The probabilistic mapping from stimulus (rock) to outcome (snake) shifted over the course of the experiment (Fig. 1c), requiring participants to track this relationship over time. When an outcome was revealed, the presence of a snake was deterministically associated with an electric shock delivered to the back of the left hand. Over the course of 320 trials, the probabilistic mapping between stimuli and outcomes changed every 26–38 trials, requiring participants to maintain and update their beliefs about the probability of a snake being under either rock. Participants chose correctly on 68% of trials on average. Our use of electric shock proved an effective elicitor of cortisol release. A Skillings–Mack test (used to account for non-normality and missing data, see Methods) confirmed that cortisol concentrations changed over the course of the experiment (Skilling's Mack *T*_7_=18.48, *P=*0.010). Paired tests indicated that this was due to an elevation of cortisol above baseline 20 min after the first shocks were received (Wilcoxon rank sum, *Z*=2.20, *P=*0.028), in line with the typical time course of endocrine responses^26^,^28^,^33^.

### Hierarchical Bayesian learning explains predictions of shock

**Figure 2:**
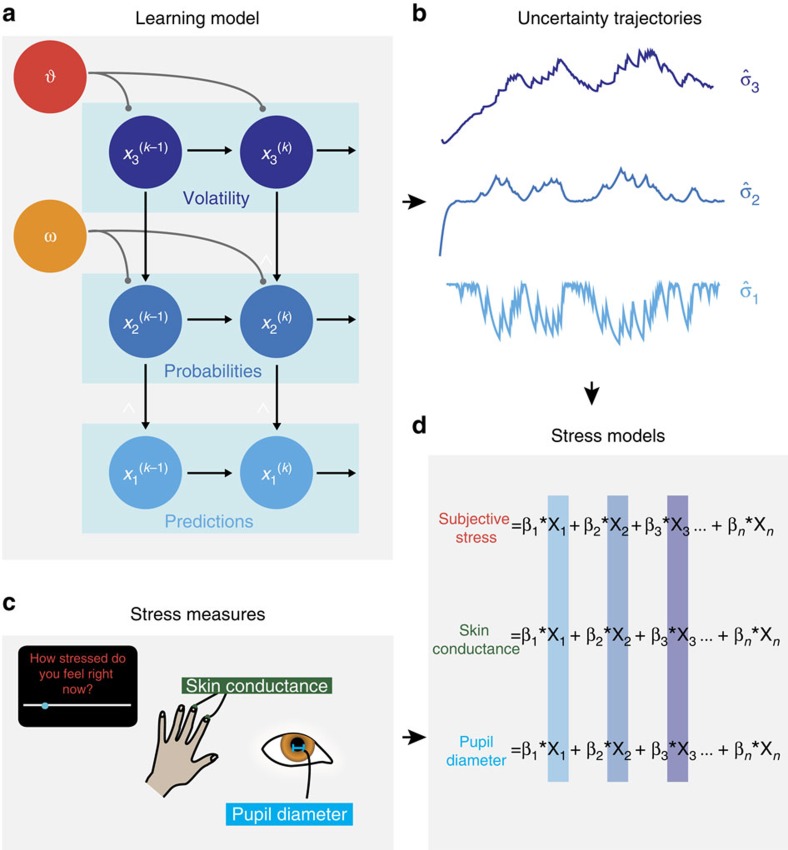
Modelling of learning and stress. (**a**) Hierarchical Gaussian Filter model^21^. Beliefs are represented in probability distributions organized in a hierarchy, with the speed of updating at each level influenced by the estimate at the level above. This allows learning to occur more quickly in volatile environments. Each level is Gaussian, characterized by a mean (*μ*) and a variance (*σ*), which corresponds to uncertainty. These representations unfold over time, with the model furnishing an estimate at each level, for each trial. We take these dynamic representations of uncertainty from this model (**b**) and use them to predict stress responses (**c**) using linear modelling (**d**). The resultant regression coefficients (*β*_1−*n*_) quantify the influence of each form of uncertainty on that stress measure. Note that X_1−*n*_ are the regressors in the linear model, which include but are not limited to uncertainty trajectories; we also include terms such as number of shocks, and nuisance variables such as gaze co-ordinates.

We compared the performance of three learning models in explaining the predictions that participants made on each trial, defining our model space by reference to a recent study using a similar prediction paradigm^15^. The simplest was a Rescorla–Wagner model^34^, in which beliefs are updated by prediction errors with a fixed learning rate. Our second model, the Sutton K1 (ref. 35), allows the learning rate to vary as a function of recent prediction errors. The third model was a three-level Hierarchical Gaussian Filter (HGF)^21^ in which beliefs are updated via prediction errors, with learning rates influenced by uncertainty about the veracity of current beliefs and environmental stability (Fig. 2a). The HGF is hierarchical in the sense that learning occurs simultaneously on multiple levels. We consider a three-level model, as this has been shown to describe learning in a similar task where tone–picture associations were learned in a non-stressful context^15^.

The first level of the HGF constitutes participants' predictions for each trial, the second level represents beliefs about probabilities that give rise to those predictions, and the third level quantifies the estimated volatility of the probabilities. On each trial, the model provides an estimate for each level, before the outcome is revealed and the estimate updated accordingly. Circumflexes (^) are used to distinguish the pre-update estimates from the updated versions. The model is Gaussian in that predictions at each level are represented by a Gaussian distribution, described by its mean, 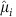, and variance, 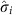, with *i* denoting the level in question (1, 2 or 3 in our model). The variance 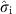 represents the uncertainty of the estimate at each level. As previously alluded to, the first-, second- and third-level variance (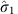,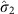 and 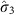) correspond to irreducible, estimation and volatility uncertainty, respectively. Updates of beliefs at each level occur via prediction errors that propagate upwards and are weighted by the ratio of the uncertainty of the level that generated them to the uncertainty of the level being updated, a form of precision weighting^15^.

**Figure 3:**
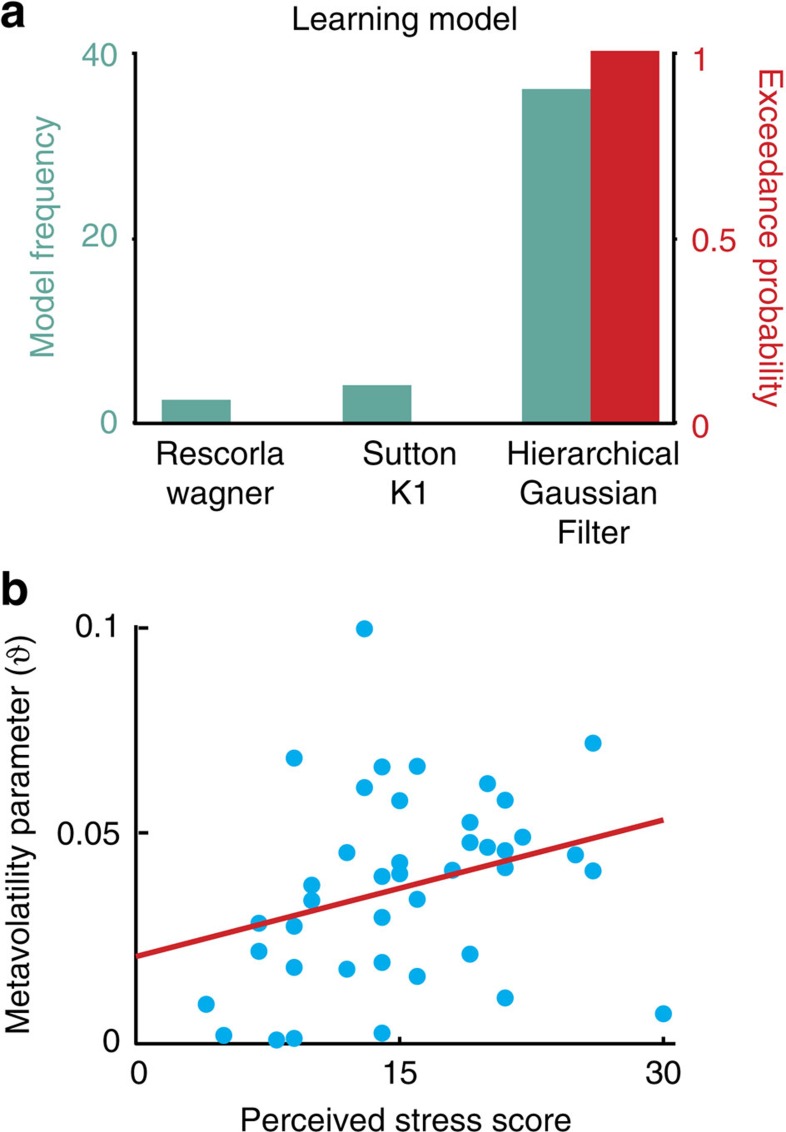
Assessing models of learning. (**a**) Random-Effects Bayesian Model Comparison confirmed that the Hierarchical Gaussian Filter (HGF) outperformed fixed-learning-rate models (Rescorla–Wagner) and variable-learning-rate non-Bayesian models (Sutton K1). (**b**) Life stress was assessed with a Perceived Stress Scale^38^. Life stress scores were correlated with the metavolatility parameter (*ϑ*) in the HGF, suggesting that more stressed individuals believe the world to be less stable (*n*=41; Spearman *ρ*=0.38, *P=*0.014).

We compared these three models (Rescorla–Wagner; Sutton K1; HGF) in a Random-Effects Model Comparison^36^, using tools freely available online^37^ (Fig. 3a). We found that the HGF was the best model by a considerable margin (model frequency=82%, exceedance probability ∼1). This is a close replication of the aforementioned study in which tone–picture associations were learned in a non-stressful context^15^. Having ascertained that the HGF was the model that best explained the predictions made by our participants, we proceeded to examine the distribution of fitted model parameters across the population. Fitting of the HGF allows for variance between individuals^15^,^21^, which in this instantiation is expressed by two parameters: *ω* and *ϑ*. *ω* is a constant component of the learning rate at the second level, capturing variability in how rapidly people update their beliefs. *ϑ* determines the rate of update of the third level; this parameter can be understood as capturing ‘metavolatility', with higher values implying a belief in a less stable world (Fig. 2a).

We found that individuals' metavolatility parameter correlated with levels of chronic stress, as assessed by a questionnaire measure of life stress, the Perceived Stress Scale^38^ (Fig. 3b; Spearman *ρ*=0.39, *P=*0.014; non-parametric statistics used due to non-normality of *ϑ* (Kolmogorov–Smirnov test, *P<*0.001)). This suggests that people who report higher levels of life stress behave as if they believe that the environment is more uncertain, indicating that chronic stress levels may be affected by prior exposure to environments of high uncertainty. This confirms that interindividual variability in stress relates to variability in beliefs about uncertainty, as expected if stress responses are tuned by exposure to uncertainty in the real world. Having established a relationship between beliefs about uncertainty and a static subjective measure of chronic stress, we next addressed the coupling between uncertainty trajectories and dynamic stress measures.

### Current irreducible uncertainty predicts subjective stress

**Figure 4:**
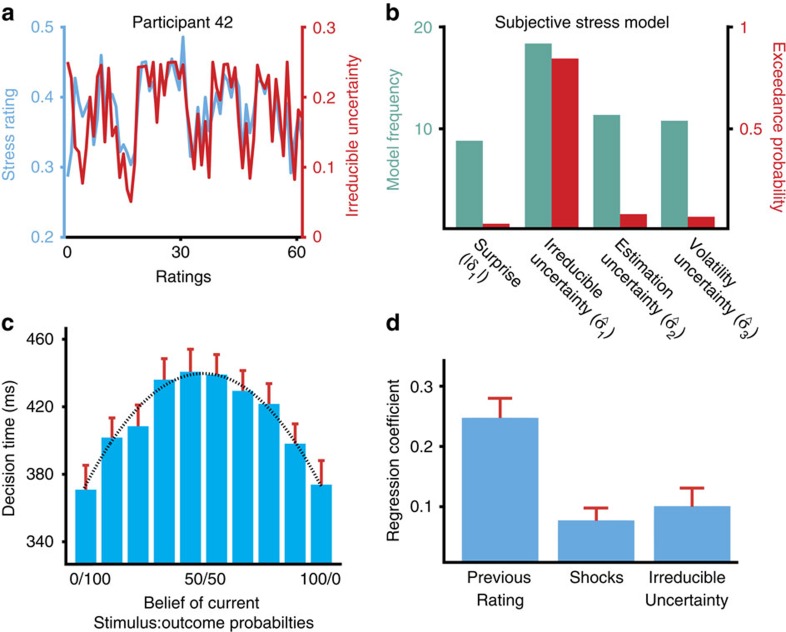
Irreducible uncertainty predicts subjective stress. (**a**) Example subjective stress trajectory for one participant (blue) and irreducible uncertainty estimates for that individual (red). (**b**) Comparison of regression models of subjective stress. All models shared two components: the value of the previous rating and the number of shocks delivered since the last rating. The surprise model summed the surprise (|*δ*_1_|) for each outcome since the previous rating, while models *σ*_1–3_ included the estimated uncertainty at each level of the Hierarchical Gaussian Filter at the time of rating. Irreducible uncertainty (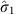) provided the best fit to our participants (*n=*45). (**c**) Irreducible uncertainty predicts prediction response times. A curve describing the variance of a Bernoulli distribution representing beliefs about probabilities, corresponding to irreducible uncertainty, predicts average response times (Pearson *r=*0.99, *P<*0.001). (**d**) The winning regression model predicting subjective stress responses (mean *r*^2^=0.25). Shocks and irreducible uncertainty both predicted subjective stress ratings (single-sample *t*-tests, *P<*0.001; *P=*0.0024). Error bars represent s.e.m.

Having found that the HGF was the appropriate model of learning in our task, we asked how the dynamic quantities represented in this model related to acute stress responses. In fitting the HGF to each participant, we obtained estimated trajectories of surprise (absolute prediction errors) and uncertainty over time (Fig. 2b). To assess the influence of surprise and uncertainty on subjective stress, we fit multiple regression models (Fig. 2d) to subjective stress ratings for each participant (example trajectory shown in Fig. 4a). We compared the ability of four different models to predict stress ratings. All four models incorporated the previous stress rating and the number of shocks received since the last rating. Our predictions therefore took the following form:


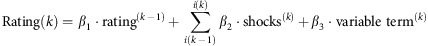


Here *k* represents the rating number (1–65), and *i(k)* is the trial number (1–320) associated with rating *k* (ratings occurred on average every 4–6 trials). Hence, the shock term is the number of shocks received since the last subjective stress rating.

Our first model summed the surprise experienced by a participant since the last rating, as captured by the variable *δ*_1_ in the HGF. The other three regression models quantified the uncertainty represented at each level of the HGF: irreducible uncertainty (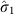), estimation uncertainty (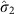) and volatility uncertainty (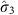).

Subjective stress responses were best predicted by a model incorporating solely the current level of irreducible uncertainty (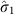; model frequency=41%, exceedance probability=0.849; Fig. 4b). The resultant model is depicted in Fig. 4d. As predicted, participants reported being most stressed when they believed the current state was high in irreducible uncertainty. All parameters were significantly greater than zero (single-sample *t*-tests on parameters from multiple regression: previous rating *β*=0.25, *t*_44_=7.60, *P<*0.001; shocks *β*=0.074, *t*_44_=3.58, *P<*0.001; irreducible uncertainty *β*=0.099, *t*_44_=3.22, *P=*0.0024). We found no evidence for a model of subjective stress featuring multiple forms of uncertainty (Supplementary Fig. 1A). In addition, we found that the estimates of subjective irreducible uncertainty furnished by the HGF provided better predictions of subjective stress than the objective irreducible uncertainty on each trial (Supplementary Fig. 1B,C).

Subjective irreducible uncertainty is highest in our task when the subject's estimated probability of a shock is 50%, corresponding to a situation where the environment is utterly unpredictable, and maximal in entropy^17^,^18^. There is an inverted-U relationship between irreducible uncertainty and probability, according to the variance of a Bernoulli distribution (uncertainty=probability × (1−probability)). This relationship was also reflected in participants' behaviour, in that they were slowest making decisions under conditions of maximal uncertainty (Fig. 4c; Pearson correlation, *r=*0.99, *P<*0.001).

We found that subjective stress responses are predicted by the trajectory of irreducible uncertainty experienced by each individual. The link between subjective and physiological indices of stress is problematic, with proposals that stress responses should exhibit coherence^29^ not well supported by extant data^39^. Consequently, we next asked whether irreducible uncertainty also predicts physiological arousal, examining its relationship with two standard physiological stress measures, pupil diameter and skin conductance (Fig. 2c).

### Physiological stress reflects uncertainty and surprise

**Figure 5:**
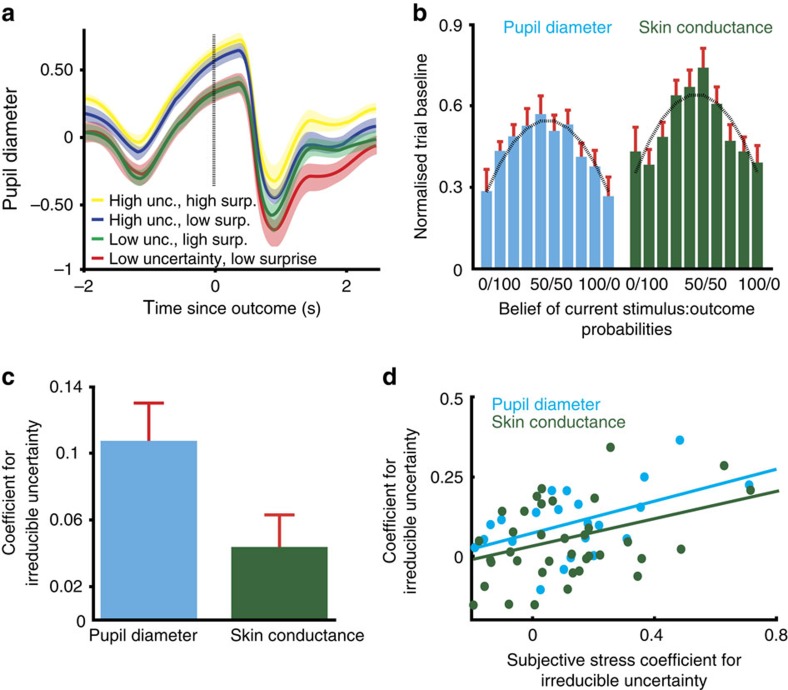
Physiological responses reflect uncertainty and surprise. (**a**) Median splits indicate that both irreducible uncertainty and surprise increase pupil diameter (both *P<*0.001). Shading is s.e.m. across participants. (**b**) Baseline pupil diameter and skin conductance on each trial displayed a clear inverted-U relationship with belief, as seen for subjective stress. A curve describing the variance of a Bernoulli distribution fit well (Pearson correlations: pupil diameter: *r=*0.96, *P<*0.001; skin conductance: *r=*0.84, *P=*0.002). Error bars represent s.e.m. (**c**) A multiple regression model demonstrated a role for uncertainty throughout the trial for both pupil diameter (robust regression *β*=0.11, single-sample *t*-test, *t*_21_=4.72, *P<*0.001) and skin conductance (robust regression *β*=0.044, single-sample *t*-test, *t*_36_=2.25, *P=*0.031). Error bars represent s.e.m. (**d**) Across subjects, the sensitivity of subjective stress to irreducible uncertainty correlated with the sensitivity observed in pupil diameter (Pearson correlation, *n=*22, *r=*0.52, *P=*0.013) and skin conductance (Pearson correlation, *n=*37, *r=*0.38, *P=*0.021) models. Each data point is one participant.

As a first step in gaining insight into the role played by uncertainty, we epoched physiological responses (pupil diameter, *n*=22; skin conductance, *n*=37; see Methods) by trial, starting 2 s before an outcome was revealed. On the basis of evidence that pupil diameter and skin conductance reflect surprise^40^, and building on our finding that subjective stress responses are predicted by irreducible uncertainty, we implemented median splits, separating trials according to whether they were high or low in irreducible uncertainty and high or low in surprise. This resulted in four groupings (high/high, high/low, low/high and low/low). Taking the average across participants, we observed that uncertainty increased pupil diameter throughout the trial (Fig. 5a), with an additional, positive effect of surprise ∼2 s after outcome presentation. The time course of skin conductance responses was similar, albeit slower (Supplementary Fig. 2A). Two-way analysis of variance demonstrated that both pupil diameter and skin conductance were increased by irreducible uncertainty (pupil: *F*_1,21_=22.56, *P<*0.001, *η*^2^=0.051; skin conductance: *F*_1,36_=9.36, *P=*0.004, *η*^2^=0.104) and surprise (pupil: *F*_1,21_=20.71, *P<*0.001, *η*^2^=0.045; skin conductance: *F*_1,36_=12.40, *P=*0.001, *η*^2^=0.070), with no interaction (pupil: *F*_1,21_=0.51, *P=*0.48; skin conductance: *F*_1,36_=0.14, *P=*0.71).

Within the framework of our model, information about uncertainty on the current trial is available to the subject before the trial begins, as it is computed on the basis of trial history^21^. Consequently, we asked whether baseline pupil diameter and skin conductance, as assessed at the start of each trial, reflected the subjective belief of probabilities on that trial, as represented in our learning model. We found that baseline arousal displayed an inverted-U relationship with belief, strikingly reminiscent of the relationship between reaction time and belief (compare Figs 4c and 5b). To confirm this relationship, we show that a curve fit to the variance of a Bernoulli distribution, describing the relationship between irreducible uncertainty and belief, captured this relationship well (Pearson correlations, pupil: *r=*0.96, *P<*0.001; skin conductance: *r=*0.84, *P=*0.002).

To examine more precisely this relationship between uncertainty, surprise and skin conductance, we employed a model-based approach (Fig. 2d), convolving response functions for pupillary^41^ and skin conductance^42^ responses with our predictors (see Methods for full details of model and Supplementary Fig. 3 for details of pupillary response function). We included surprise as a regressor in our models to ensure that responses to uncertainty were independent of the surprise at outcome (Supplementary Fig. 2B). We found that the level of irreducible uncertainty on each trial was a significant predictor of both pupil diameter (robust regression *β*=0.11, single-sample *t*-test *t*_21_=4.72, *P<*0.001) and skin conductance (*β*=0.044, *t*_36_=2.25, *P=*0.031; Fig. 5c).

Finally, we asked whether the sensitivity of physiological stress to uncertainty related to that inferred from reported subjective stress responses. We took the magnitude of the regression coefficients (*β*) for irreducible uncertainty from our models of pupil diameter and skin conductance, and compared them with the equivalent terms from our subjective stress model. In both cases, the two were positively correlated (Pearson correlation, pupil: *r=*0.52, *P=*0.013; skin conductance: *r=*0.38, *P=*0.021; Fig. 5d) such that individuals whose subjective reports were more sensitive to uncertainty also showed a greater impact of uncertainty upon their physiological stress responses. This concordance between emotional and physiological state is predicted by theories of emotion^43^, although direct evidence for this relationship is rare^44^. Our computational perspective on the cognitive dynamics of stress responses reveal a strong coherence between emotional and physiological systems, although we note that the sensitivity of the two physiological measures was not themselves correlated (Supplementary Fig. 4).

### Uncertainty tuning of stress predicts performance

**Figure 6:**
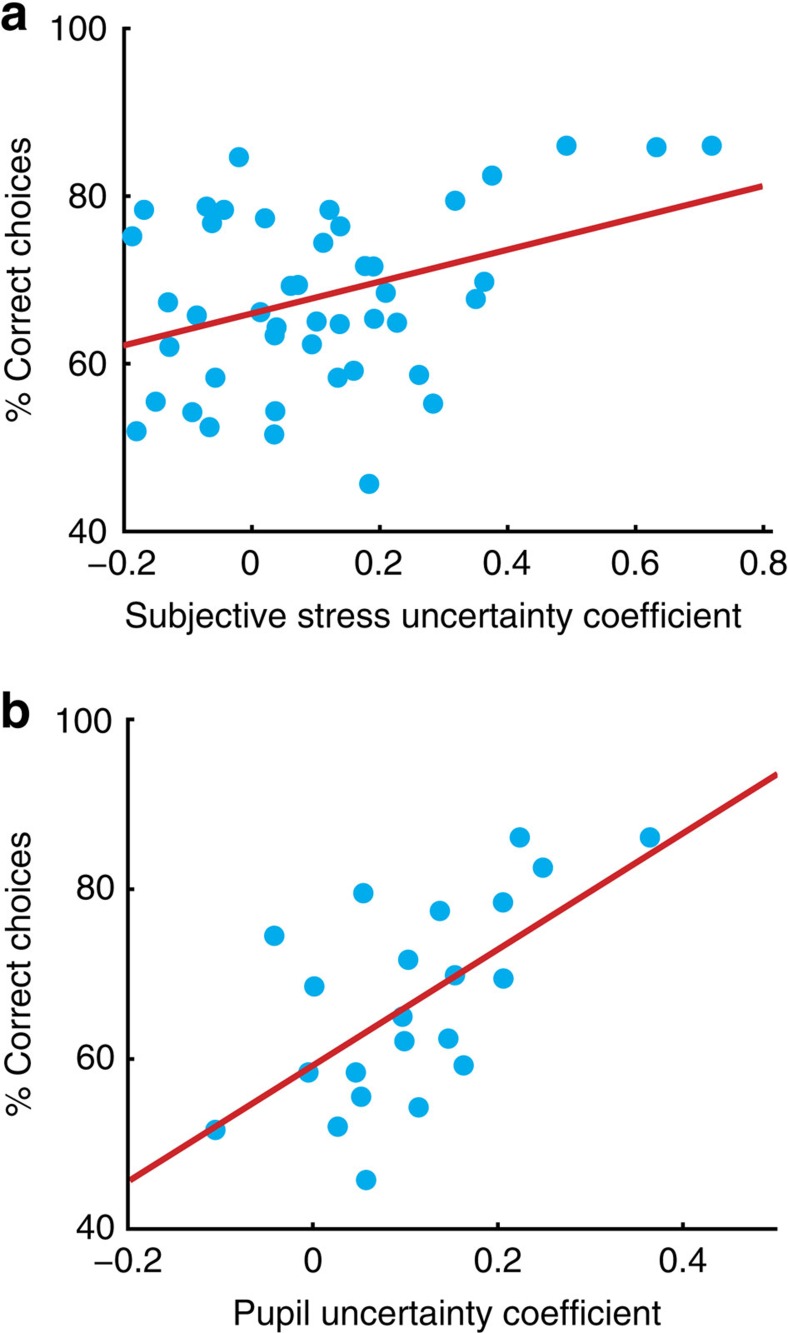
Relationship between uncertainty sensitivity and task performance. (**a**) Subjective stress sensitivity (the regression coefficient for uncertainty in the subjective stress model) correlated with how frequently participants predicted the correct outcome (Pearson correlation, *n=*45, *r=*0.37, *P=*0.012). (**b**) Pupillary sensitivity to uncertainty also predicted performance (Pearson correlation, *n=*22, *r=*0.62, *P=*0.0023). Each data point is one participant.

We hypothesized that if the tuning of stress responses to uncertainty is adaptive, the degree of coupling between uncertainty and stress responses would predict how well participants performed in our task. We found this was indeed the case, as both subjective (Pearson correlation, *r=*0.37, *P=*0.012) and pupillary (Pearson correlation, *r=*0.62, *P=*0.0023) sensitivity to uncertainty predicted task performance (Fig. 6a,b). Thus, the degree to which stress responses track irreducible uncertainty in the environment predicts learning under uncertain threat, in accordance with an adaptive account of stress responses under uncertainty. No such relationship was evident between gross measures of stress such as the mean or variance of stress ratings, highlighting the utility of our model-based approach (Supplementary Fig. 5). We also observed a negative relationship between intolerance of uncertainty and pupil diameter (Supplementary Fig. 6).

## Discussion

Stress responses are co-ordinated physiological and behavioural responses to environmental challenges^1^. The precise features of the environment that generate stress have proved hard to pin down, particularly within a quantitative framework. Here we reveal a strong relationship between stress and subjective estimates of a quantifiable property of the environment, namely, irreducible uncertainty. This demonstrates that computational models of learning can provide quantitative metrics of environmental and psychological variables that drive emotional and physiological stress responses. In the present case, this highlights a striking relationship between a specific form of uncertainty and stress responses.

We built on recent progress in computational modelling of subjective well being^45^, to inform a dissection of subjective stress responses. This was made possible by a hierarchical Bayesian model that allowed us to infer the trajectory of uncertainty experienced by each individual in our experiment. The use of computational models has proved indispensable in exploring the relationship between stress, genotype and behaviour^46^, but has not to our knowledge previously been applied to understand the genesis of stress responses. Our finding that such models can be used to link subjective uncertainty and stress responses adds to a growing consensus that detailed quantitative models are indispensable for the understanding of complex biological and mental phenomena^47^,^48^,^49^,^50^.

Having identified that irreducible uncertainty best predicted subjective stress responses, we next asked whether physiological responses were similarly predicted by uncertainty. Pupil diameter is a readout of central arousal thought to relate to noradrenergic activity in the locus coeruleus^51^. Recent evidence suggests that locus coeruleus firing correlates with pupil diameter in the macaque monkey, and that stimulation of the locus coeruleus is sufficient to induce changes in pupil diameter^52^. Although noradrenergic dynamics are crucial in orchestrating acute stress responses^53^ as well as their behavioural^54^ and mnemonic^55^ impact, pupillometry is not typically employed in studies of stress (though see Henckens *et al*. for a notable exception). This is surprising given that pupillary response metrics provide insight into emotional^31^ and cognitive dynamics^41^,^56^,^57^. Our finding that pupil diameter reflects the surprise associated with an outcome, regardless of valence, replicates previous results^56^,^57^,^58^. Furthermore, we also find a correlation between pupil diameter and the current level of irreducible uncertainty, with greater pupil diameter associated with higher levels of uncertainty. In rewarding environments, pupil diameter has been shown to reflect estimation uncertainty and, as we find here, irreducible uncertainty, often referred to as risk^56^,^57^.

A recent study examining individual differences in aversive learning found a post-outcome pupillary sensitivity to volatility^58^. The authors found that learning-rate malleability in the face of changing volatility was related to trait anxiety. We additionally establish a link between chronic stress states and beliefs of environmental volatility (Fig. 2c). However, the stimulus–outcome contingencies used in the previous study kept irreducible uncertainty roughly constant, precluding the comprehensive characterization of multiple forms of uncertainty and the relation to the dynamics of emotional and physiological stress responses that we perform here. Our finding that pre-outcome pupil diameter correlates with irreducible uncertainty, and that this modulation is proportional to the effect of uncertainty on subjective stress, is uniquely enabled by our design, and not inconsistent with the volatility sensitivity observed previously.

Fluctuations in skin conductance depend on activity in the sympathetic nervous system^42^, a key component of physiological stress responses^53^. Our finding that skin conductance tracks uncertainty chimes with findings using the Iowa Gambling Task, in which participants make choices between decks of cards that produce rewards and punishments of varying magnitudes. Greater skin conductance responses are elicited whenever participants choose a card from the pack with higher risk, as defined by the variance of the outcome distribution^59^. However, binary choices in static environments do not reveal whether skin conductance truly reflects uncertainty or instead some aspect of the decision process. Our results suggest that even in non-instrumental settings, somatic state relates closely to uncertainty in the environment. Furthermore, we show that these responses are dynamically driven by evolving internal estimates of irreducible uncertainty.

Our multiple stress measures reflect the view that stress is a multidimensional construct, expressed through subjective and physiological channels^43^. Some theoretical accounts highlight the importance of ‘coherence' between stress systems in response to a challenge^29^, although discordance between the amplitude of physiological and subjective stress responses is rife (reviewed by Campbell and Ehlert^39^). This is in part because the stressors typically used in laboratory experiments with humans, such as social stress, are difficult to parameterise, precluding a detailed quantitative analysis. Conversely, using a quantitative computational approach, we show that emotional and physiological stress responses track uncertainty and are correlated within individuals. We do not claim that ours are exhaustive metrics of stress, nor that chronic stress necessarily behaves similarly to the acute stress examined here. Establishing how acute stress accumulates to produce allostasis^60^, and what effects such allostasis exerts on subsequent acute stress responses, is a major challenge for the field.

An integrated understanding of normal brain function and its perturbation in disease will require detailed analysis at multiple levels of description, from behavioural to cellular. Here we provide a computational account of acute stress responses in humans. Dysfunction of stress response systems is common to many psychiatric disorders^2^, suggesting that a computational decomposition of stress responses of the kind provide here may prove a fruitful addition to the nascent field of computational psychiatry^61^.

## Methods

### Participants

All experiments were approved by the University College London Ethics Review Board. Participants (*n=*45, 25 females, aged 19–35 years) were recruited via the UCL Institute of Cognitive Neuroscience recruitment mailing list, and gave their written informed consent before beginning any experiments. All participants were healthy, with no history of neurological or psychiatric disorders, and no family history of epilepsy. Sample size was based on recent experiments involving stress^33^.

### Task

Participants initially underwent a shock thresholding procedure (see below). Participants then received thorough instruction (see below) that made explicit the structure of the task, and completed a practice session of 30 trials of the probabilistic learning task used in the main experiment. They were also familiarized with the use of the subjective stress rating scale.

Our learning task was closely modelled on that used in a recent study leveraging the same computational framework in a non-stressful context^15^. Timings on each trial were jittered using a uniform distribution to allow us to maximally divorce physiological responses to different events.

Each participant completed a set of 320 trials. On each trial, participants were presented with one of two stimuli (in our case, rocks). These stimuli remained on screen for 300 ms (±50 ms) before participants were asked to make a prediction, signalled with a button press, as to which outcome (snake or no snake) was likely to follow (Fig. 1a,b). This decision was made under time pressure, with a timeout period averaging 1,000 ms (±200 ms).

Once the decision had been made, the prediction was displayed for an average of 1,200 ms (±200 ms), before the outcome was revealed. Outcomes remained on screen for 1,000 ms (±200 ms). In the case of the snake stimulus, outcome presentation was coincident with the delivery of a shock. This was followed by an intertrial interval of 2,000 ms (±500 ms), during which a fixation cross was displayed.

The probabilistic mapping from stimulus to outcome shifted over the course of the experiment (Fig. 1c), requiring participants to constantly track the relationship over time. This resulted in fluctuations in the level of various forms of uncertainty. Each session of 320 trials was divided into 10 blocks of different stimulus–outcome probabilities, of lengths that varied between 26 and 38 trials. The transitions between these blocks were not made explicit to the subject. The probabilities governing each block varied from heavily biased (90/10), through moderately biased (70/30) to unbiased (50/50), allowing us to examine the effect of predictability on stress responses. We used four repeats each of the biased probability blocks (2 for each bias direction, that is, 70/30 and 30/70) and two repeats of the 50/50 to generate 10 blocks in total.

Participants were paid a base rate of £10 and informed that they would receive an extra £0.05 for each correct prediction they made. Outcomes (correct/incorrect) were not explicitly signalled. Participants were allowed to take a self-timed break every 10 min.

For 41 of the 45 subjects, we report questionnaire measures of life stress (Perceived Stress Scale, PSS). Data from the remaining participants were lost due to a technical error. Some subjects (*n=*23) completed questionnaires on a separate day, while the remainder (*n=*22) did so after the main task. We also collected a questionnaire measure of intolerance of uncertainty (*n=*43; Supplementary Fig. 6), depression (Beck Depression Index), and a questionnaire related to anxiety (Mood & Anxiety Symptom Questionnaire). We do not report data from the latter two questionnaires here.

### Participant instruction

Participants were given detailed computerized instruction on the structure of the task. This emphasized that the accuracy of their predictions did not affect the number of shocks they received but did influence their earnings on the task. Understanding was confirmed with the question: ‘How much do you earn per correct prediction?'

We also attempted to ensure good understanding of the probabilistic relationships governing stimulus:outcome relationships. We emphasized that the probabilities were reciprocal (p(snake|rock A)=1−p(snake|rock B)), and checked for comprehension with the question: ‘If the probability of a snake being under rock A is 40%, what is the probability of it being under rock B?'

Participants were further informed that the probabilities changed throughout the task, and that at times might appear to be random, that is, the probability of an outcome following each stimulus might be equal (50/50).

### Shock thresholding procedure

Electric shocks were controlled using a Digitimer DS5 system in conjunction with a National Instruments Data Acquisition Board, which allowed control of shock amplitude via Matlab (Mathworks). Electrodes were placed 0.5 cm apart on the first dorsal interosseous of the left hand. Electrode sites were cleaned with alcohol and a mild abrasive (NuPrep Skin Prep Gel). Shocks were delivered using BioPac Ag/AgCl electrodes filled with Sigma Spectra 360 Electrode Gel, attached using double-sided adhesive pads.

Participants first underwent a thresholding procedure that allowed us to map their subjective sensitivity to shock. Thresholding consisted of a sequence of 80 shocks, with currents of magnitudes between 0.1 and 10 mA chosen according to a staircasing procedure. After each shock, participants were asked to report how painful it was, from a rating of 1 (not painful) to 5 (very painful). We used an automated thresholding procedure inspired by Gracely *et al*.^62^, in which separate staircases are used to estimate the transition points between each rating (1/2,2/3,3/4,4/5). For robustness, we used two independent staircases, running the QUEST thresholding algorithm^63^ for each transition. This gave a total of eight independent staircases, with trials from each staircase randomly interleaved. At the end of thresholding, we averaged the two estimates for the 4/5 boundary to set the shock intensity for the rest of the experiment. Participants were given a sample shock at this intensity and in three cases we reduced the amplitude of the experimental shock by 20% at the participant's request. Shock sensitivity was also measured at the end of the task, and on average showed a slight decrease (single-sample *t*-test, *t*_44_=2.70, *P=*0.097, *d*=0.403), equivalent to an 11% reduction in subjective pain.

### Stress measures

Each participant was asked to make 65 subjective stress ratings at semiregular points throughout the probabilistic learning task (every 4–6 trials). These required participants to move a marker along a line to answer the question ‘How stressed do you feel at this moment?' (Fig. 1d). All analyses were conducted on *z*-scored ratings to obviate between-subject differences in use of the scale.

Pupil diameter was recorded in a subset of participants (*n=*22) using an EyeLink 1000 System (SR Research), sampled at 100 Hz. Participants were seated in a darkened room, and asked to maintain fixation wherever possible. Stimuli were luminance matched, with the no snake outcome signalled by a scrambled version of the snake picture.

Skin conductance was recorded from the index and middle fingertips of the left hand using 8 mm BioPac AgCl electrodes. Electrodes were filled with a 0.5%-NaCl paste (BioPac Gel 101) and attached using double-sided adhesive pads supplemented by tape. We utilized a custom recording system based on the provision of a constant current between the two electrodes and the measurement of the resultant voltage, allowing calculation of the conductance of the skin (AT64, Autogenic Systems). This signal was converted to an optical pulse and then digitally recorded at 100 Hz in Spike2 (v6.17).

In a subset of subjects (20), we collected saliva samples at 8 time points, from which we measured cortisol concentrations. To avoid baseline elevation due to anticipatory stress we collected two baseline readings (samples 1 and 2) on a separate day, on which participants were aware that no shocks would be received. On the day of the experiment, we collected the following samples: (3) on arrival; (4) immediately before task (∼15 min after shock thresholding); (5) 10 min into task; (6) 20 min into task; (7) 30 min into task; and (8) post task. Participants salivated through straws into 2-ml polypropylene tubes. Samples were frozen on the day of collection. Analysis was performed by Viapath at King's College Hospital, using a competitive immunoassay. Briefly, cortisol in the sample competes with cortisol conjugated to horseradish peroxidase for binding sites on a microtitre plate. Unbound reagents are then washed away. Bound cortisol enzyme conjugate is measured by the reaction of the horseradish peroxidase enzyme to the substrate tetramethylbenzidine, producing a blue colour. A yellow colour is formed after stopping the reaction with an acidic solution. The concentration of cortisol in the sample is calculated as a function of the optical absorption at 450 nm; more absorption implies greater concentration of cortisol enzyme conjugate, and therefore lower concentration of cortisol in the sample. For further details, see Arakawa *et al*.^64^. Some samples were not suitable for analysis due to damage in storage (28/160). To accommodate for these missing values, we used a Skilling–Mack test to assess changes in cortisol over time. One subject was excluded due to baseline concentrations >3 s.d.'s away from the mean (60.59 nmol l^−1^; population mean=6.92 nmol l^−1^; population s.d.=13.57 nmol l^−1^). To verify that this did not affect our conclusions, we repeated our analyses without excluding this subject, and found comparable results. All data were log transformed before analysis to render data close to normal^33^.

### Analysis of pupil diameter

Data were exported using the EDF2ASC plugin, and imported as ASCII files into Matlab. Pupil diameter measurements were downsampled to 100 Hz, and low-pass filtered (4 Hz, third-order Butterworth)^41^. Blinks were automatically detected by the EyeLink software, and removed by linear interpolation of samples 140 ms either side of the blink. Data were then *z*-scored and detrended. No further artefact removal was necessary.

Linear modelling involved a mixture of delta and boxcar regressors convolved with a canonical pupillary response function (see below for details of response functions)^41^. For phasic responses, we also convolved regressors with the first and second derivatives of the canonical response function, a standard method in magnetic resonance imaging^65^ designed to accommodate inaccuracies in the modelling of the amplitude and timing of induced responses. These were subsequently orthogonalised to their respective regressors, to apportion shared variance to the primary regressor. We took an additional step to remove signal due to changes in luminance, which produces pupil constrictions discernible from emotional/cognitive pupillary changes by their short latency^31^. We estimated a luminance response function for each subject on the basis of passive viewing of the images in our task (each image presented 50 times, displayed for 1,000 ms, with a jittered intertrial interval of between 9,000 and 1,100 ms). This provided a response function that could then be convolved with each presentation of an image, allowing us to discriminate fast, luminance-dependent constrictions from slower dilatations relating to cognitive variables (Supplementary Fig. 3). Following convolution of predictors with their response functions, predictors were *z*-scored. Details of the full linear model can be found in Supplementary Table 1.

### Analysis of skin conductance

Data were first visually inspected and eight participants were rejected due to low recording quality.

Data from the remaining 37 participants were imported and preprocessed using tools from the SCRalyze suite^42^. Data were downsampled to 10 Hz and low-pass filtered (5 Hz, first-order Butterworth). We used a custom artefact rejection regime based on the second differential of the signal; non-physiological signals were identified by their very rapid rate of change. Subjects took self-paced breaks every 10 min, which caused substantial changes in skin conductance amplitude due to movement of the arm, and so we discarded the first five trials following a break. The time series was then concatenated, detrended and *z*-scored.

For linear models, we used a mixture of delta and boxcar regressors to represent phasic and sustained influences on skin conductance. These regressors were then convolved with the canonical skin conductance response function outlined in ref. 35 and provided in SCRalyze (http://scralyze.sourceforge.net/) (see below for details of response functions). We utilized a variety of nuisance regressors to isolate changes in skin conductance relating to our cognitive variables of interest. All predictors were *z*-scored before modelling. Details of the full linear model can be found in Supplementary Table 2.

### Modelling of learning

We modelled learning in our task using several models. Three of these (Rescorla–Wagner, Sutton K1, Hierarchical Gaussian Filter) were implemented using the HGF toolbox (http://www.translationalneuromodeling.org/hgf-toolbox-v3-0/). For a full list of priors, see Supplementary Table 3. For details of each model, see Supplementary Table 4.

The model that best explained our data was the HGF (Fig. 2a). Introduced by Mathys *et al*.^21^, the HGF is a Bayesian learning model not constrained by the optimality typically assumed by such models; instead, subject-specific fitting allows for interindividual variability in learning. A recent functional magnetic resonance imaging study using the HGF highlighted its utility in assessing learning under uncertainty and its neural correlates^15^; our task and analysis were inspired by the ones used there. For a full description of the structure of the HGF, the reader is referred to the start of the Results section.

The HGF was fit to each individuals' choices using Variational Bayes, with two free parameters: *ϑ*, a metavolatility parameter that determines step size at the third level of the HGF; and, *ω*, which is a constant component of the learning rate at the second level.

The four quantities utilized in our analyses of stress measures are all trajectories over time, with a value that evolves according to the predictions made and outcomes experienced by that subject.

The first of these is surprise (|*δ*_1_| in the HGF). This is the difference between the observed outcome (1=snake /0=no snake) on trial *k* and the subject's belief about the probability of that outcome:


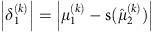


where *μ*_1_^(*k*)^ is the actual outcome (1 or 0) and *s*(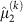) is the sigmoid transformation of belief about probabilities before seeing the outcome, that is, the subject's expectations. By taking the absolute value of *δ*_1_^(*k*)^, we therefore obtain a quantity that represents surprise about outcomes.

The three forms of uncertainty we consider are as follows:


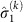
: uncertainty of predictions at the first level on trial *k*. Because beliefs at the first level take the form of a Bernoulli distribution, the variance is a function of the mean 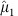, namely, 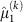
_ ×_ (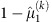). This means that uncertainty has an inverted-U relationship with belief, as depicted in Figs 3c and 4b. Intuitively, this form of uncertainty represents an individual's estimate of the entropy of the environment at that moment in time; that is, how surprising they expect things to be. We refer to it as irreducible uncertainty.


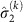
: this is a form of informational uncertainty on trial *k*, representing lack of knowledge about the current stimulus:outcome relationship. Over time and in a stable environment, this uncertainty would fall to zero as the probabilities underlying the task are learned. In volatile environments, however, this is not the case. In the HGF, this form of uncertainty is approximately equivalent to a time-varying learning rate, used to update beliefs quickly when they are uncertain and slowly when they are supported by plentiful evidence. We refer to it as estimation uncertainty.


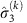
: this can also be considered a form of estimation uncertainty, this time over the volatility of the environment at trial *k*. Again, it controls the speed of learning about volatility, weighting prediction errors from the probability space at the second level. We refer to it as volatility uncertainty.

We ran an additional pair of Rescorla–Wagner learning models to test the validity of two assumptions made by the models in the original comparison. The first is that we assume participants update probabilities symmetrically on each trial: if p(outcome|stimulus 1) increases then p(outcome|stimulus 2) decreases by the same amount, as constrained by our task and explained to participants. To accommodate for departures from this scheme, we used a Rescorla–Wagner model in which the probabilities for each stimulus were updated independently. Second, we examined the possibility that beliefs were updated differently following trials on which shocks were delivered by fitting two learning rates (*α*_shock_ and *α*_noshock_) for each subject. We then compared these two models to the original Rescorla–Wagner model (in which probabilities were updated symmetrically with a single learning rate). Bayesian Model Comparison showed that the simple model comprehensively outperformed the two variants (exceedance probability=1). We concluded, therefore, that the assumptions of symmetric probability updating and balanced learning across shock/no shock trials were justified.

### Modelling of stress

We used multiple regression models to examine the relationship between task variables, including the trajectories from the HGF outlined above, and stress responses. We used least-squared error to fit data from subjective ratings, using Matlab function glmfit. For physiological data, we used robust fitting to avoid spurious fits due to unidentified artefacts. These were implemented in Matlab with the function robustfit. In both cases, we used two-tailed *t*-tests to assess whether parameters were different from zero, that is, whether, at the population level, a given parameter meaningfully and consistently contributed to the dependent variable in question.

We compared several multiple regression models to examine the effect of uncertainty on subjective stress. All models included parameters for the previous rating and the number of shocks received since the last rating. The third term varied between models, and captured the effects of absolute prediction error (model 1), and uncertainty at each level (models 2–4).

*Model 1: surprise*. Model 1 omitted an explicit representation of uncertainty, but summed the absolute prediction errors (|*δ*_1_|, bounded 0–1 on each trial) since the last rating, reflecting surprise in response to outcomes given an individual's beliefs.

*Model 2: irreducible uncertainty*. Irreducible uncertainty is the variance of the Bernoulli distribution representing subject's beliefs, captured by the HGF parameter 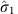. It is highest when p(outcome|stimulus 1) and p(outcome|stimulus 2) are both equal to 0.5, that is, the sequence of outcomes is totally unpredictable. Irreducible uncertainty is also correlated with the magnitude of surprise (surprise is on average higher in uncertain situations).

*Model 3: estimation uncertainty*. Uncertainty about the probabilities currently governing the observed outcomes is known as estimation uncertainty. This is represented in the HGF by the variance of the Gaussian distribution representing beliefs at the second level, 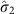.

*Model 4: volatility uncertainty*. Finally, we tested the hypothesis that subjective stress related to uncertainty at the third level, corresponding to uncertainty about the volatility of the generative process. Again, this is explicitly represented in the HGF as the variance of the Gaussian representing beliefs at the third level, 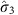.

For physiological stress measures, we convolved predictors with canonical response functions (see below) to account for the time course of physiological responses^41^,^42^.

### Model comparisons

We performed two sets of model comparisons. In the first, we determined the best learning model to explain predictions made by the subjects, and in the second the best model for subjective stress responses. In each case, for each model and each subject, we took the model evidence (*F*-values for learning models or Bayesian Information Criterion^66^ for multiple regression models), and used these to assess model fit by Random-Effects Bayesian Model Selection^36^, as implemented in the VBA toolbox^37^. Random-Effects Bayesian Model Selection allows for heterogeneity in the population; the best model for each individual is allowed to vary, producing an estimate of model frequency in the population (that is, for how many participants that model is the best model) and an exceedance probability (the probability that the model in question is the most frequently utilized in the population).

### Response functions used in pupillary and skin conductance measures

We used linear modelling to elucidate the impact of different events on pupil diameter and skin conductance. This approach is well-established in the functional magnetic resonance imaging literature, where the use of General Linear Model (GLM) to interpret haemodynamic responses is common.

In this approach, a response function is used to describe how the output of a system (the measured variable) relates to its inputs. This response function is then convolved with a representation of the putative inputs to the system, and the resultant time course is used as a predictor in the GLM. A common further step is to create secondary and tertiary regressors that are convolved with the first and second derivative of the response function^65^. These ‘dispersion regressors' allow for inaccuracy in the timing or form of modelled responses; we utilize this approach in our modelling of events (this is unnecessary when the modelled process is extended over time, and represented by a boxcar).

Our response functions were drawn directly from previous studies. For skin conductance, we used the skin conductance response functions provided in SCRalyze (http://scralyze.sourceforge.net/), which are based on a gamma function convolved with a Gaussian kernel^67^.

Response functions for pupillary dilatation were first discussed by Hoeks *et al*.^68^ and we use the response function described there, which takes the form of a gamma function:


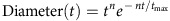


The constants *n* and *t*_max_ dictate the shape of the response function. As advocated in the original paper, we use values of *n*=10.1 and *t*_max_=930 ms; we note that a recent study using this response function^41^ demonstrated that this algorithm is robust to changes in the values of the constants used (see the Supplementary Information in ref. 41).

We took an additional step in our modelling of pupillary responses. As the appearance of stimuli and outcomes involved increases in luminance, they evoked light-related pupil constrictions. Importantly, such luminance responses are faster than the dilatations evoked by cognitive or emotional factors^31^. We accounted for light-related constrictions by convolving stimulus and outcome onsets with a luminance response function, which was fitted to each subject on the basis of a separate data set in which subjects were passively exposed to each of the image used in our experiment (see above and Supplementary Fig. 3). The best fitting parameters were found using least-squared fitting implemented by the Matlab function fmincon. Calculated in this way, average *n*=3.6 and *t*_max_=839 ms.

### Statistical analysis

All data analysis was completed in Matlab (Mathworks). All statistical tests were two-sided. We used one-sample *t*-tests to test for the significance of parameters in multiple regression models, and two-way repeated-measures analysis of variance to analyse the time-course data for skin conductance and pupil diameter. We used Pearson correlation coefficients except in the one case in which the data were *a priori* not normal, having been fit in a logit space (*ϑ*; Fig. 2c). In this instance, we confirmed non-normality using a Kolmogorov–Smirnov test, and used Spearman's Rank to assess the correlation non-parametrically.

### Code availability

Custom Matlab code for analysis of skin conductance and pupil diameter is available on request to the corresponding author. We used the HGF toolbox (http://www.translationalneuromodeling.org/hgf-toolbox-v3-0/) for modelling of learning, the VBA toolbox for model comparison (mbb-team.github.io/VBA-toolbox/), and the SCRalyze suite for preprocessing of skin conductance data (http://scralyze.sourceforge.net/).

## Additional information

**How to cite this article:** de Berker, A. O. *et al*. Computations of uncertainty mediate acute stress responses in humans. *Nat. Commun.* 7:10996 doi: 10.1038/ncomms10996 (2016).

## Supplementary Material

Supplementary Information Supplementary Figures 1-6, Supplementary Tables 1-4, and Supplementary References
