# Supplementary material for: Computations of uncertainty mediate acute stress responses in humans
Source: Nat Commun. 2016 Mar 29;7:10996. doi: 10.1038/ncomms10996 (PMC4820542; doi:10.1038/ncomms10996)
Supplement: Supplementary Information — Supplementary Figures 1-6, Supplementary Tables 1-4, and Supplementary References [file ncomms10996-s1.pdf]

SUPPLEMENTARY FIGURES

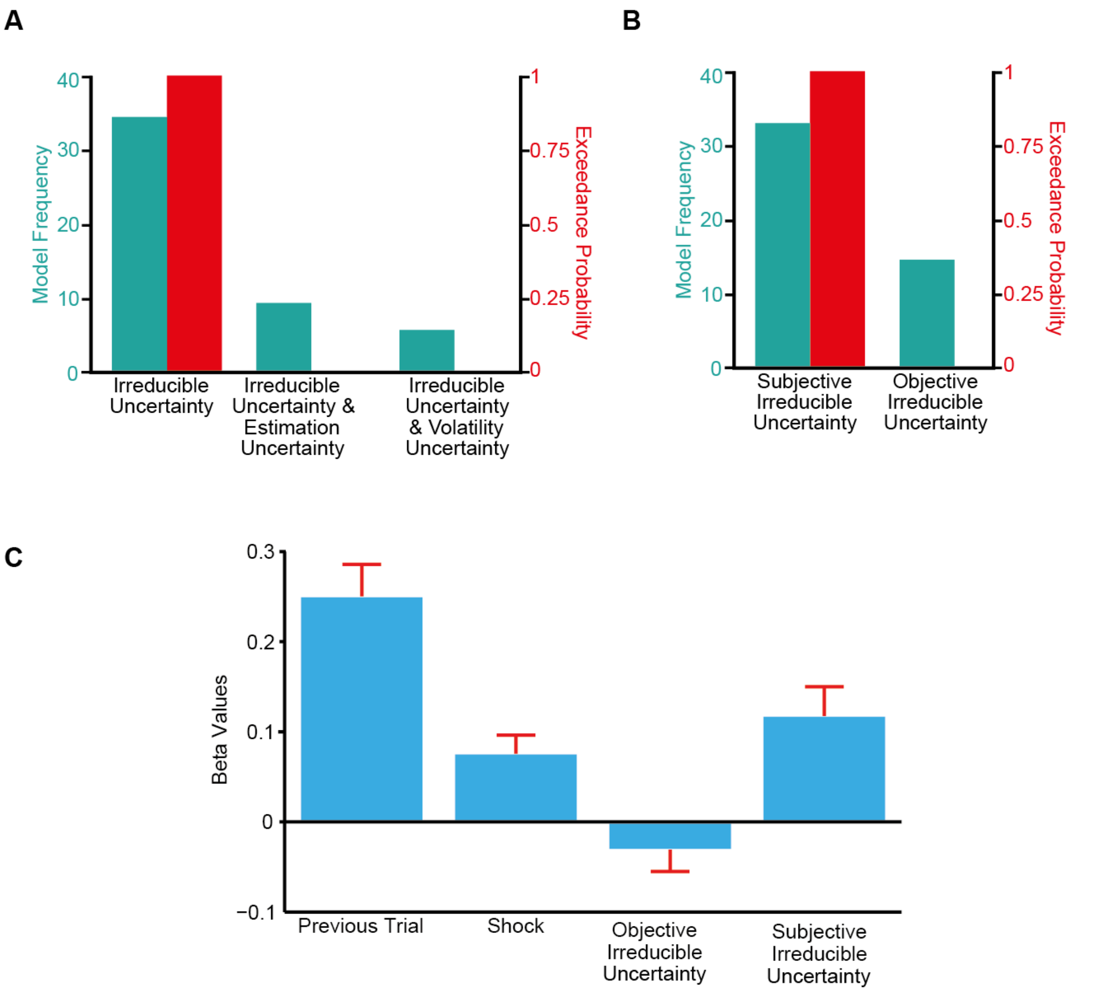

Supplementary Figure 1

Assessing alternative models of subjective stress (A) Model comparison demonstrated that adding additional estimation or volatility uncertainty did not improve the performance of the irreducible uncertainty model used to explain subjective stress responses (Model Frequency=70.5%, Exceedance Probability~1). (B) Model comparison confirmed that subjective uncertainty as furnished by the HGF model provided a better predictor of subjective stress (Model Frequency=70.5%, Exceedance Probability=0.993) than objective uncertainty. (C) As expected, objective and subjective uncertainty are correlated (Pearson correlation, mean *r*=0.452); if both are placed in the model, only subjective uncertainty provides a significant predictor of subjective stress. Error bars are SEM across participants.

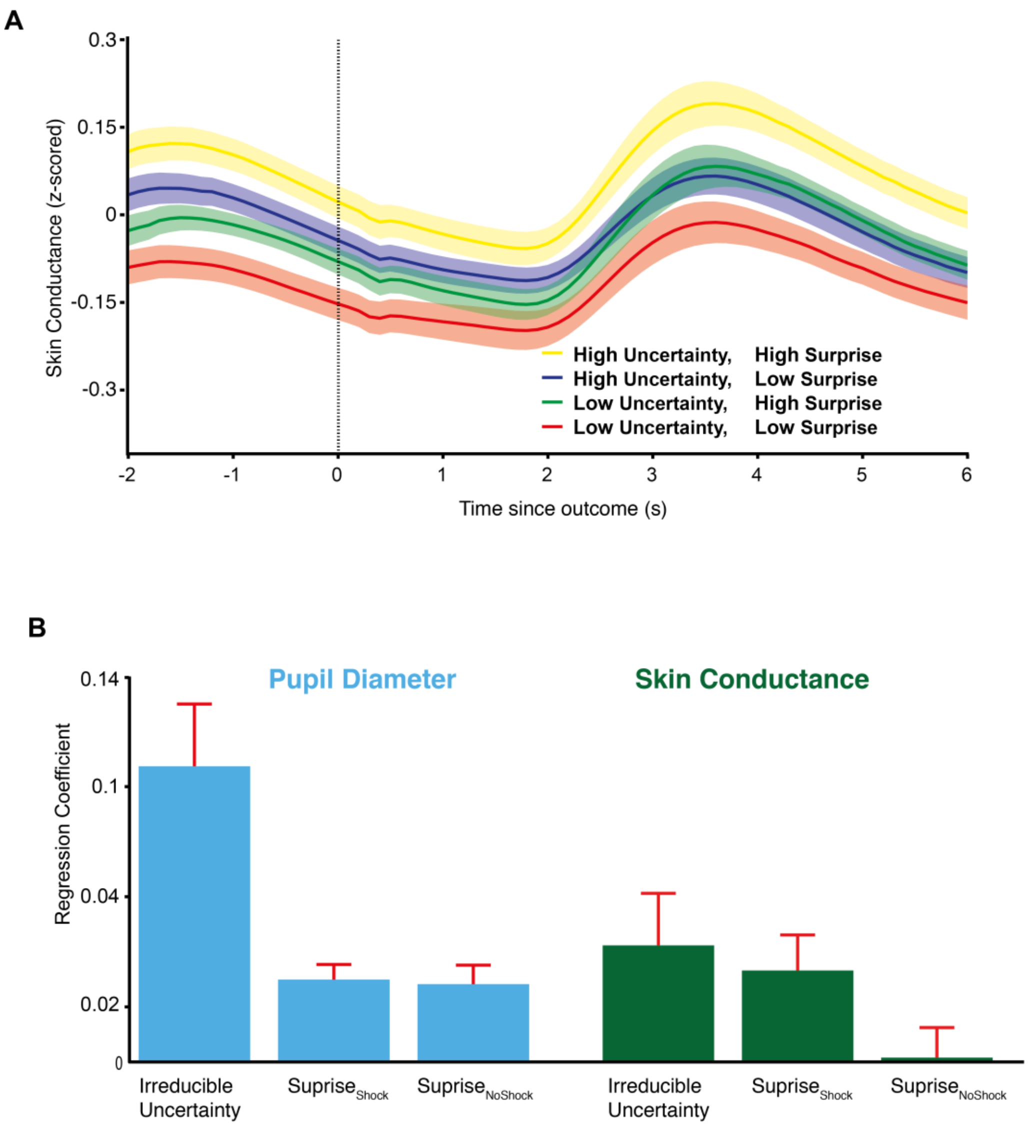

Supplementary Figure 2

Additional physiological stress data (A) We split trials by uncertainty and surprise (see main results and Fig. 4A). Average skin conductance was higher for trials with higher uncertainty and surprise (Repeated Measures ANOVA, Uncertainty: $F_{1,36}$=9.36, p=0.004, η^2=0.104, Surprise: $F_{1,36}$=12.40, p=0.001, η^2=0.070), with no evidence of an interaction ($F_{1,36}$=0.14, p=0.71). (B) Our regression models of pupil diameter and skin conductance included regressors for surprise at outcome,to ensure that correlations with surprise were not attributed to uncertainty. Uncertainty was a significant predictor of both pupil diameter (robust regression, β=0.11, single-sample t-test t_{21}=4.72, p<0.001) and skin conductance (robust regression, β=0.044, single-sample t-test t_{36}=2.25, p=0.031), with surprise predicting pupil diameter equally on shock

and no shock trials (robust regression, $Surprise_{Shock}$: β=0.018, single-sample t-test t_{21}=4.11, p<0.001; $Surprise_{NoShock}$: β=0.017, t_{21}=3.05, p=0.0060; Difference, paired t-test t_{21}=0.209, p=0.84). However, skin conductance reflected surprise asymmetrically, with the parameter for surprise on shock trials greater than that for no-shock trials (robust regression, $Surprise_{Shock}$: β=0.035, single-sample t-test t_{36}=2.58, p=0.014; $Surprise_{NoShock}$: β=0.0019, single-sample t-test t_{36}=0.167, p=0.87; Difference, paired t-test t_{36}=1.84, p=0.07). This replicates previous observations of asymmetric prediction error representations in skin conductance measurements [1]. However, a direct comparison of parameters from participants for whom we recorded both pupil diameter and skin suggested that the two were not significantly different, with neither a main effect (Repeated Measures ANOVA, Effect of recording modality, $F_{1,35}$=2.82, p=0.11, η^2=0.025) nor an interaction between modality and regressor (Repeated Measures ANOVA, Modality x Regressor interaction, $F_{1,35}$=0.96, p=0.39, η^2=0.012). We are therefore unable to reject the null hypothesis that pupil diameter and skin conductance track aversive learning in comparable manners; disparities between the two may therefore be a result of low signal-to-noise in skin conductance measurements. Error bars are SEM across participants.

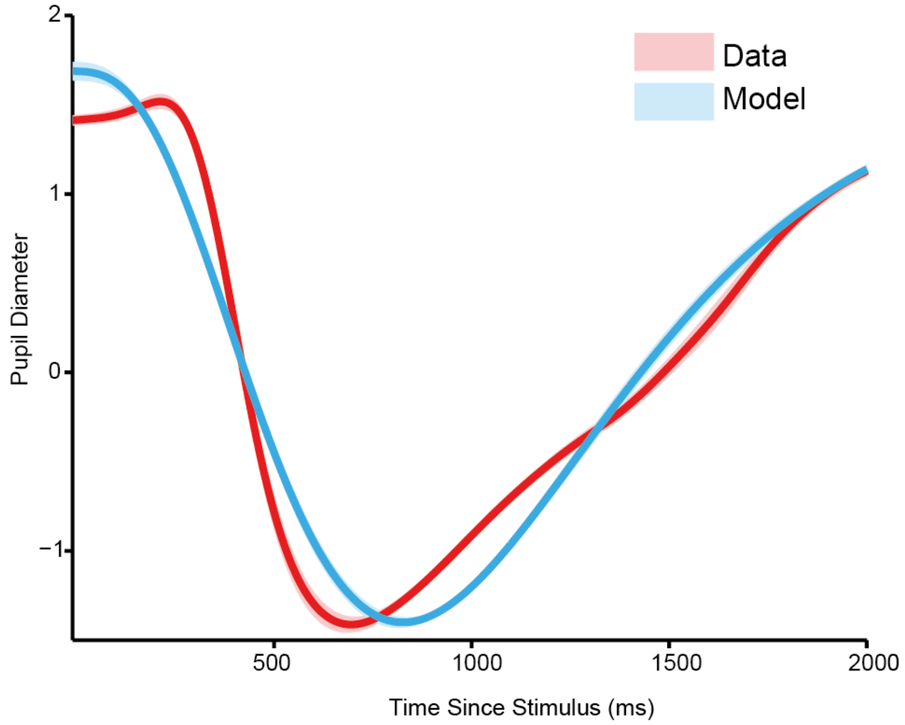

Supplementary Figure 3

Luminance fitting procedure used for model of pupil diameter. In order to accommodate fluctuations in pupil diameter induced by luminance changes, we fit a luminance response function for each subject. This was conducted using a reference data set acquired after the experiment, in which each of the images used in the experiment was shown 50 times. We then fit a single luminance response function using data from all image presentations (red trace above, shading is SEM). We used the gamma response function defined in [2] which has two free parameters: time to peak (T_{max}) and number of cascade components (n). We found the best fitting pair of parameters for our luminance response function using gradient descent methods implemented by the MatLab function fmincon (blue trace above, shading is SEM). As expected from the fast constriction typically associated with the light reflex, the average T_{max} in our luminance response function was smaller than that used in the conventional response function (839ms vs 930ms). We were thus able to include luminance responses in our regression models of pupil diameter (Figure 5 and S2B).

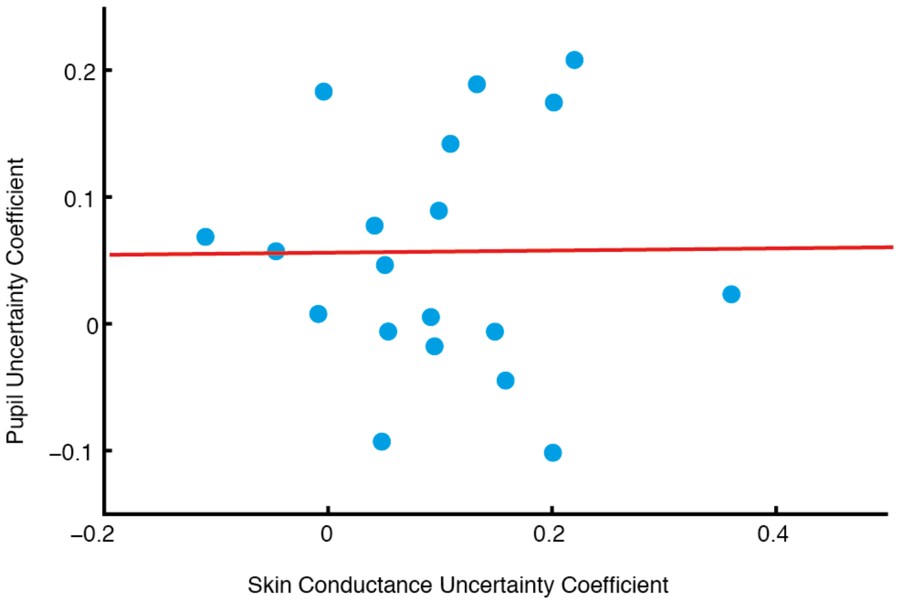

Supplementary Figure 4

Pupillary and skin conductance sensitivity to uncertainty are uncorrelated. We found no evidence of correlation between pupillary and skin conductance sensitivity to uncertainty (Pearson correlation, n=19, r=0, p=0.97). This is in contrast to the positive correlation observed between each of these parameters and subjective stress uncertainty sensitivity (Figure 5D).

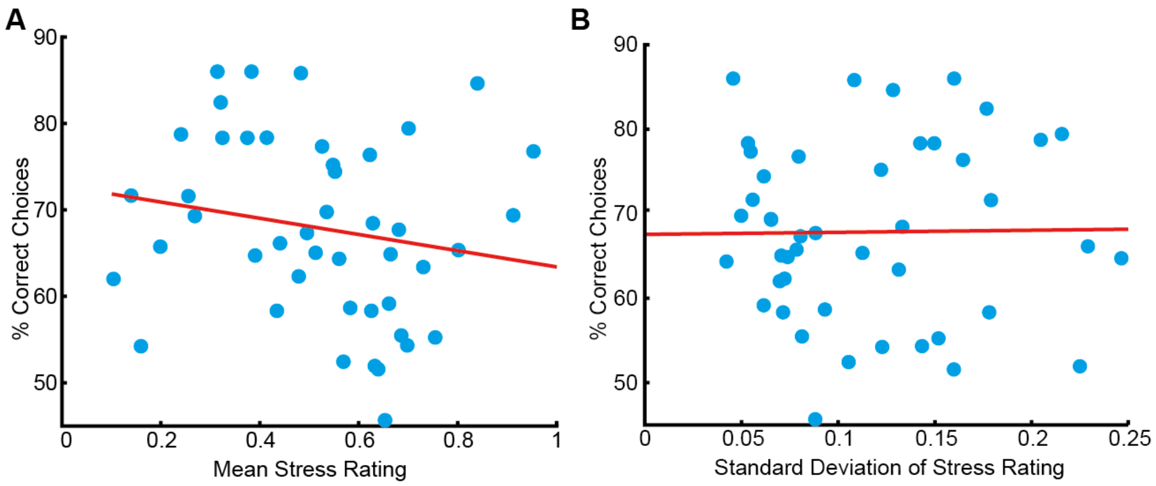

Supplementary Figure 5

Mean and variance of subjective stress ratings are unrelated to performance. Neither mean (A) (Pearson correlation, n=45, r=-0.18, p=0.23) nor standard deviation (B) (Pearson correlation n=45, r=0.014, p=0.93) of subjective stress ratings relate to performance on our task. Conversely, computational modelling reveals that the ‘uncertainty-tuning’ of stress responses based on dynamic uncertainty estimates is correlated with task performance (6e 5).

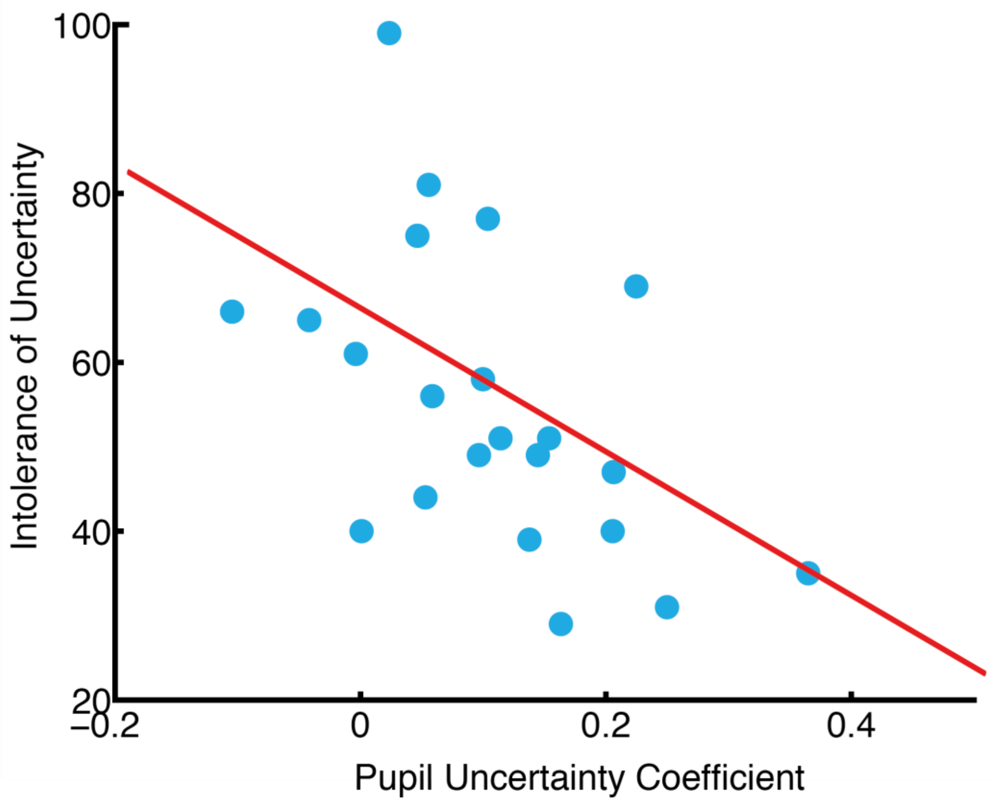

Supplementary Figure 6

Uncertainty-tuning in the pupil is inversely correlated with Intolerance of Uncertainty. Pupillary sensitivity to uncertainty was inversely related to a questionnaire measure of uncertainty aversion (Intolerance of Uncertainty; Buhr et al., 2002). Subjects with greater pupil sensitivity to uncertainty were less averse to uncertainty (Pearson correlation, n=22, r=-0.51, p=0.015). This may be related to the other effect we observed, that performance under uncertainty is predicted by the sensitivity of the pupil to uncertainty (Figure 6B); aversion to uncertainty may be a preference rooted in the fact that individuals whose pupils do not track uncertainty perform poorly when in uncertain situations.

SUPPLEMENTARY TABLES

Supplementary Table 1: Parameters used in pupil model

**Parameter**	**Mean**	**SEM**	t_{21}	***p***	**Form**
Constant	0.0614	0.0229	2.68	0.0140	
Stimuli*	-0.0704	0.0165	-4.27	<0.001	Delta function, aligned to stimuli presentations, luminance convolved
Outcome	-0.6922	0.1774	-3.90	<0.001	Delta function, aligned to all outcomes, luminance convolved
Shocks	0.4930	0.1042	4.73	<0.001	Delta function aligned to shock outcomes
No Shocks	0.2561	0.1106	2.32	0.0308	Delta function aligned to no shock outcomes
Surprise $_{Shock}$	0.0183	0.0045	4.11	<0.001	Delta function aligned to shocks and scaled by trial surprise ($\|\delta_1\|$)

Surprise $_{\text{No Shock}}$	0.0167	0.0055	3.05	0.0060	Delta function aligned to no shocks and scaled by trial surprise ($\lvert\delta_1\rvert$)
Irreducible Uncertainty	0.1069	0.0227	4.72	<0.001	Boxcar from stimulus onset, scaled by trial irreducible uncertainty (σ_1)
Gaze X coordinate	-0.0501	0.0102	-4.90	<0.001	Unconvolved
Gaze Y coordinate	0.0051	0.0218	0.23	0.818	Unconvolved

Predictors were convolved with a standard pupillary response function (see SI Methods).The exceptions were the gaze X and Y coordinates, which were unconvolved, and the Stimuli regressor. For all phasic responses (Stimuli, Outcome, Shock, No Shock, Surprise Shock, Surprise No Shock), we also convolved predictors with first and second derivatives of the response function to allow for variance in the shape and timing of the response (data not shown).

* The Stimuli regressor was not convolved with the canonical pupillary response function, but with the luminance response function estimated for each subject. See Supplementary Figure 3 for details.

Supplementary Table 2: Parameters used in skin conductance models

Parameter	Mean	SEM	t_{21}	*p*	Form
Constant	-0.1054	0.0111	-9.47	<0.001	
Stimuli	-0.0093	0.0087	-1.07	0.2905	Delta function, aligned to stimuli presentations, luminance convolved
Outcome	0.1177	0.0260	4.53	<0.001	Delta function, aligned to all outcomes, luminance convolved
Shocks	0.0698	0.0242	2.89	0.0066	Delta function aligned to shock outcomes
No Shocks	-0.1890	0.0347	-5.45	<0.001	Delta function aligned to no shock outcomes
Surprise Shock	0.0347	0.0134	2.59	0.0139	Delta function aligned to shocks and scaled by trial surprise (|δ1|)

Surprise No Shock	0.0019	0.0113	0.17	0.8686	Delta function aligned to no shocks and scaled by trial surprise (|δ1|)
Irreducible Uncertainty	0.0442	0.0196	2.25	0.0305	Boxcar from stimulus onset, scaled by trial irreducible uncertainty (σ1)

Predictors were convolved with a standard skin conductance response function (see SI Methods).

We also included regressors for each block (i.e. each stretch of 10 minutes between breaks) to account for changes in baseline between blocks.

As with the pupil model, for all phasic responses (Stimuli, Outcome, Shock, No Shock, Surprise Shock, Surprise No Shock), we also convolved predictors with first and second derivatives of the response function to allow for variance in the shape and timing of the response.

Supplementary Table 3

Parameter	Notes	Value
Model constants		
ϑ	Metavolatility parameter, controlling step size at the third level. Estimated in logit space.	**Mean** = 0<br>**Variance** = 16
ω	Constant component of the learning rate at the second level. Estimated in native space.	**Mean** = -2<br>**Variance** = 16
κ	Modulates coupling between 3rd and 2nd levels. Held constant.	
Trajectories<br>Note that since uncertainty ($\boldsymbol{\sigma}$) has a natural lower bound at zero – one cannot have negative uncertainty – it is estimated in log space. The numbers given here refer to values in that space.		
**Predictions (X_1)**	The predictions are a sigmoid transformation of the probabilities represented in X_2, and so do not have a starting value.	$\hat{\boldsymbol{\mu}}_{\mathbf{1}}$:<br>**Mean** = none<br>**Variance** = none<br>$\hat{\boldsymbol{\sigma}}_{\mathbf{1}}$:<br>**Mean** = none<br>**Variance** = none
**Probabilities (X_2)**	A starting value of 0 implies neutrality between outcomes. Starting variance was chosen to be Bayes optimal using the tools	$\hat{\boldsymbol{\mu}}_{\mathbf{2}}$:<br>**Mean** = 0

	provided in the TAPAS toolbox ('tapas_bayes_optimal_binary_config').	**Variance** = 0
		$\hat{\sigma}_2$: **Mean** = 0.06 **Variance** = 0
**Volatility (X_3)**	The absolute starting value of X_3 is arbitrary, as changes in fitted parameters will affect scaling.	$\hat{\mu}_3$: **Mean** = 1 **Variance** = 0
		$\hat{\sigma}_3$: **Mean** = 4 **Variance** = 0

Supplementary Table 4: Details of each learning model used

Model	Notes	Estimated parameters: mean (standard deviation)
Rescorla-Wagner	Beliefs are symmetrically updated, with a learning rate fitted to each subject.	α=0.38 (0.21)
Asymmetric Rescorla-Wagner	Beliefs are asymmetrically updated, with beliefs about the two stimuli updated individually.	α=0.47 (0.25)
Dual Learning Rate Rescorla-Wagner	Beliefs are updated with different learning rates on shock and no shock trials; two learning rates fitted to each subject.	α_{Shock}=0.36 (0.24)<br>$\alpha_{NoShock}$=0.35 (0.24)
Sutton K1	Beliefs updated with a variable learning rate that depends upon the amplitude of recent prediction errors.	μ =1.65 (3.00)<br>ν=0.53 (0.31)<br>h=0.005 (0.002)
HGF	Three layer model with two fitted parameters governing connections between layers and step size at the top layer.	θ=0.034 (0.02)<br>ω=-2.80 (2.43)

Supplementary References

1. Bach, D. R. & Friston, K. J. No evidence for a negative prediction error signal in peripheral indicators of sympathetic arousal. *Neuroimage* **59,** 883–884 (2012).
2. de Gee, J. W., Knapen, T. & Donner, T. H. Decision-related pupil dilation reflects upcoming choice and individual bias. *Proceedings of the National Academy of Sciences* **111,** E618–25 (2014).
